# Thymic regeneration and tumor immunotherapy

**DOI:** 10.3389/fimmu.2026.1891681

**Published:** 2026-08-25

**Authors:** Zengkuan Chen, Qi Wang, Qiqi Lu, Weiwei Liu, Xianguo Wu

**Affiliations:** Department of Laboratory Medicine, The Second Affiliated Hospital of Zhejiang University School of Medicine, Hangzhou, China

**Keywords:** thymic epithelial cells, thymic involution, thymic regeneration, thymus, tumor immunotherapy

## Abstract

The thymus, as a vital immune organ in the human body, provides the essential microenvironment for the development, differentiation, and maturation of T lymphocytes. With advancing age, the thymus gradually undergoes atrophy and degeneration, primarily characterized by structural disruption of thymic epithelial cells, leading to slowed T cell development and reduced output. Experimental studies have identified multiple regenerative pathways, including IL-7, KGF, BMP4, IL-22, RANKL, regulatory T cells, FOXN1-directed approaches, and sex-steroid blockade. Recent human observational studies also associate preserved thymic health with lower disease risk and better outcomes after cancer immunotherapy. However, most regenerative interventions remain preclinical, and no thymus-directed strategy has yet been shown to improve cancer immunotherapy outcomes in a randomized clinical trial. This review summarizes mechanisms of thymic involution and injury, evaluates regenerative strategies according to evidence level, and discusses the opportunities, limitations, and safety considerations for translating thymic regeneration into cancer care.

## Introduction

Immunosenescence refers to the gradual decline in immune system function with age, mainly manifested as deficits in both innate and adaptive immunity. A key feature is thymic involution, characterized by the progressive shrinkage and degeneration of the thymus with age, leading to arrested T cell development, reduced output, and diminished immune responsiveness. This is closely associated with the occurrence and development of opportunistic infections, autoimmune diseases, and tumors. This section summarizes the function of the thymus, as well as the consequences and mechanisms of thymic involution.

## Thymic involution

### Structure and function of the thymus

The thymus is the site for T lymphocyte development and differentiation. Hematopoietic progenitor cells from the bone marrow enter the thymus and primarily undergo three developmental stages: the CD4^-^CD8^-^ double negative (DN) stage, the CD4^+^CD8^+^ double-positive (DP) stage, and the CD4^+^ or CD8^+^ single-positive (SP) stage. During the DN stage, newly entered hematopoietic progenitor cells move into the cortex and migrate towards the subcapsular region. During this process, they undergo commitment to the T cell lineage, rearrangement and selection of the TCRβ chain genes. In the DP stage, cells migrate deeper into the cortex, completing TCRα chain gene rearrangement, positive selection, and differentiation into CD4^+^ and CD8^+^ T cells. During the SP stage, cells migrate into the medulla, undergoing negative selection, functional maturation, and divergent differentiation into Tregs. Ultimately, they export a highly diverse T cell repertoire capable of distinguishing “self” from “non-self” to the periphery (1, 2).

Besides T cells, another crucial cell type in the thymus is the thymic epithelial cell (TEC), which plays an irreplaceable role in T cell development, differentiation, and selection (3, 4). Based on anatomical structure, TECs are classified into cortical TECs (cTEC) and medullary TECs (mTEC). cTECs specifically express Ly51 and CD205. Their main functions include: 1) secreting CXCL12 and CCL25 to recruit hematopoietic progenitor cells into the thymus; 2) highly expressing Notch ligands (DLL4) to induce lineage commitment of progenitors towards the T cell lineage; 3) secreting IL-7 and SCF to promote T cell survival and proliferation; 4) expressing components involved in antigen processing and presentation, including the thymoproteasome subunit (β5t), cathepsin (CTSL), and thymus-specific serine protease (TSSP), mediating positive selection during T cell development. mTECs specifically express UEA-1. Their main functions include: 1) secreting CCL21 to promote T cell migration to the medulla; 2) mature mTECs, driven by Aire, can ectopically express tissue-restricted antigens (TRAs), mediating negative selection (5); 3) they can promote the differentiation of CD4^+^ T cells into CD25^+^Foxp3^+^ Treg cells through agonist selection. While cTECs and mTECs were once considered internally homogeneous cell populations, recent studies indicate they are highly heterogeneous. Single-cell sequencing studies (6) show that cTECs include DLL4^hi^ and DLL4^lo^ subpopulations, while mTECs also consist of CD80^lo^Aire^-^MHCII^lo^ and CD80^hi^Aire^+^MHCII^hi^ populations. These phenotypically diverse cell groups play indispensable roles in the T cell development process.

### Age-related thymic involution

Although the thymus plays an irreplaceable role in T cell development, its volume continuously decreases with age, accompanied by structural disruption and functional decline—a physiological phenomenon known as thymic involution. Morphologically, the functional areas composed of epithelial cells and developing T cells shrink, while the perivascular spaces occupied by fibroblasts and adipocytes increase. Functionally, there is a reduction in the number of DP thymocytes and a significant decline in the capacity to export T cells. Past research suggested thymic involution begins after puberty, but the process actually starts from the first year of life, declining at ~3% per year until age 35-45, after which it slows to ~1% per year (7). A study using an RAG2-GFP mouse aging model showed that total mouse thymocytes peak at 6 weeks and then begin to decline; by 6 months, total thymocytes are less than 10% of the peak, and recent thymic emigrants subsequently decrease (8). Under conditions of reduced T cell output, to maintain relatively stable numbers of peripheral T cells in the internal environment, memory T cells undergo homeostatic proliferation, causing the proportion of naive T cells to continuously decrease, ultimately leading to reduced diversity of the T cell receptor repertoire. Furthermore, the phenotype and function of T cells produced by these aged thymi also change, mainly characterized by upregulation of the senescence marker CD57 and reduced proliferative capacity upon antigen stimulation (9). The overall consequence of these changes is a diminished ability to respond to novel antigens, vaccines, and tumor-associated antigens (10).

Additionally, thymic involution contributes to the occurrence and development of autoimmune diseases in aged individuals. In the thymus, immature T cells reactive to self-MHC molecules are typically deleted through negative selection. The thymic epithelial cells and dendritic cells involved in this negative selection are lost with age, impairing the capacity for central tolerance regulation and making it more likely that self-reactive T cells are released into the periphery (11).

### Causes of thymic involution

Age-related thymic involution can be largely attributed to three factors: hematopoietic progenitor cell senescence, thymic microenvironment defects, and hormonal changes. T cells develop from bone marrow-derived progenitors that enter the thymus, first differentiating into early T-lineage progenitors (ETPs), and then progressively differentiating (12). Although the number of hematopoietic stem cells in the bone marrow increases with age, their potential to differentiate towards the lymphoid lineage significantly declines (13). Therefore, while the homing capacity of hematopoietic progenitors remains unchanged, the reduction in lymphoid-primed progenitor numbers directly leads to a significant decrease in ETPs within the aged thymus (14). Besides numerical defects, ETPs in the aged thymus also exhibit reduced proliferative and differentiative potential. Specifically, the development of DN cells is affected, including differentiation blocks at the DN2, DN3, and DN4 stages and an abnormal increase in the CD3^+^ CD44^+^ CD24^-^ DN subpopulation. Although the precise role of this population remains unclear, similar subpopulations have been found in adult mouse bone marrow, primarily associated with declining hematopoietic function. Additionally, decreased CD3 levels in aged DP and SP thymocytes may impair TCR signaling pathways and result in diminished proliferative capacity upon antigen stimulation (15).

However, studies have shown that ETPs from aged mice can differentiate into T cells of normal number and function when placed in a young mouse thymus, suggesting thymic involution is also influenced by the microenvironment, particularly defects in thymic epithelial cells (16). With increasing age, the phenotype and proportion of thymic stromal cell subpopulations change significantly. The ratios of epithelial cells to fibroblasts, and medulla to cortex cells, are significantly lower in 52-week-old mice compared to 4-week-old mice (17). However, other research indicates that the reduction in TEC numbers in the aged thymus is not dramatic; the most significant change might be the decrease in cTEC projections (18). Beyond quantitative changes, TECs also exhibit phenotypic and functional alterations. FOXN1, a key transcription factor for TEC development and differentiation, shows decreased expression with age. In mouse models, inducing FOXN1 expression has been shown to delay age-related thymic involution (19) and rejuvenate the aged thymus by expanding the TEC compartment (20). Furthermore, thymic epithelial cells exhibit an age-related decrease in MHC class II molecule expression, diminishing their ability to interact with developing thymocytes (17). Concurrently, the cytokines secreted by TECs partly support T cell development. While IL-7 levels do not significantly decrease in the aged thymus, some pro-inflammatory cytokines like IL-6 and LIF show marked increases. Exogenous administration of IL-6 family cytokines like LIF and IL-6 leads to rapid thymic involution (21). Other studies suggest that activated T cells re-entering the thymus might also play a significant role in thymic involution by potentially inducing exhaustion of thymic epithelial progenitors (22). The overall consequence of these structural changes is the progressive loss of thymic architecture, hindering thymocyte development.

Furthermore, hormonal changes, particularly the decline in growth hormone and increase in sex hormones, are strongly correlated with thymic involution, with the most pronounced effects during puberty (23). Sex hormones, especially androgens, not only directly induce thymocyte apoptosis (24) but also influence thymic function through other mechanisms. Studies in androgen-resistant castrated mice indicated that the primary mediators of androgen-induced thymic involution are non-hematopoietic stromal cells (25). Subsequent research found that knocking out the androgen receptor in thymic epithelial cells partially relieved androgen-induced thymic involution, further implicating TECs as critical targets of androgens (26). Transcriptomic analysis revealed that androgen-dependent signaling suppresses the expression of various genes related to epithelial cell development and function, such as *Foxn1*, *Ccl25*, and *Dll4*, although the exact mechanisms promoting thymic involution remain unclear (27). Additionally, androgens diminish the differentiation capacity of hematopoietic stem cells towards the lymphoid lineage by affecting hematopoietic stem cells and the bone marrow microenvironment, resulting in a reduced number of hematopoietic progenitors entering the thymus (28).

### Thymic damage by cytoreductive therapies and aging

The thymus is the central lymphoid organ responsible for generating a diverse and self-tolerant T cell repertoire, yet it is highly vulnerable to damage from cytoreductive therapies and physiological aging, both of which drive thymic atrophy and compromised adaptive immunity.

Cytoreductive interventions, including total-body irradiation and chemotherapy, cause acute thymic injury with depletion of thymocytes and disruption of thymic epithelial cells (TECs) (29, 30). Conditioning for hematopoietic cell transplantation (HCT) can produce prolonged deficits in *de novo* T-cell reconstitution that persist for a year or longer (31). Post-transplant T-cell deficiency is associated with infectious complications (31, 32), tumor relapse (30), and secondary malignancies (33, 34). Although the thymus has intrinsic regenerative capacity, inflammatory pathways can constrain recovery. Damage-induced pyroptosis activates caspase-1 and matures interleukin-18 (IL-18), which stimulates resident cytotoxic natural killer cells. Irradiated TECs downregulate MHC class I and upregulate the NKG2D ligand RAE-1, increasing susceptibility to perforin- and granzyme-dependent killing (29). Conversely, Genah et al. identified proteasome inhibition as a route to increase Foxn1 expression: nitazoxanide accelerated structural and cellular recovery after irradiation in mice without detectable disruption of thymocyte selection (35). This pharmacological result remains preclinical.

Aging further exacerbates thymic dysfunction and restricts regenerative potential. During the aging process, the thymus undergoes significant lipoatrophy, marked by the ectopic accumulation of lipids and fatty acid byproducts. The channeling of these ectopic lipids into nonoxidative metabolic pathways leads to the generation of ceramides and other “lipotoxic DAMPs” (damage-associated molecular patterns). These lipotoxic signals serve as a critical mechanistic bridge between metabolic dysfunction and structural thymic demise by triggering local innate immune-sensing pathways (36). Kousa et al. reported that during age-related involution, atypical age-associated TECs (aaTECs) emerge and form high-density, thymocyte-depleted epithelial clusters in the peri-medullary region (37). These aaTECs exhibit partial epithelial-to-mesenchymal transition, reduced expression of the master TEC transcription factor FOXN1, and sequester trophic signals such as FGF and BMP, diverting regenerative cues away from functional TEC subsets (37). Following cytoreductive injury, aaTEC populations expand substantially, further blunting thymic recovery in aged hosts (37). Two 2025 studies further identified a local FGF21 axis in mouse thymic aging: increasing FGF21 in TECs or adipocytes preserved TEC abundance, limited fibroadipogenic remodeling, and improved thymic output, whereas disrupting FGF21 signaling in Foxn1+ TECs accelerated age-related decline (38, 39). These findings extend the earlier FGF21 work (36) but remain preclinical.

### Strategies for thymic rejuvenation

**Figure 1:**
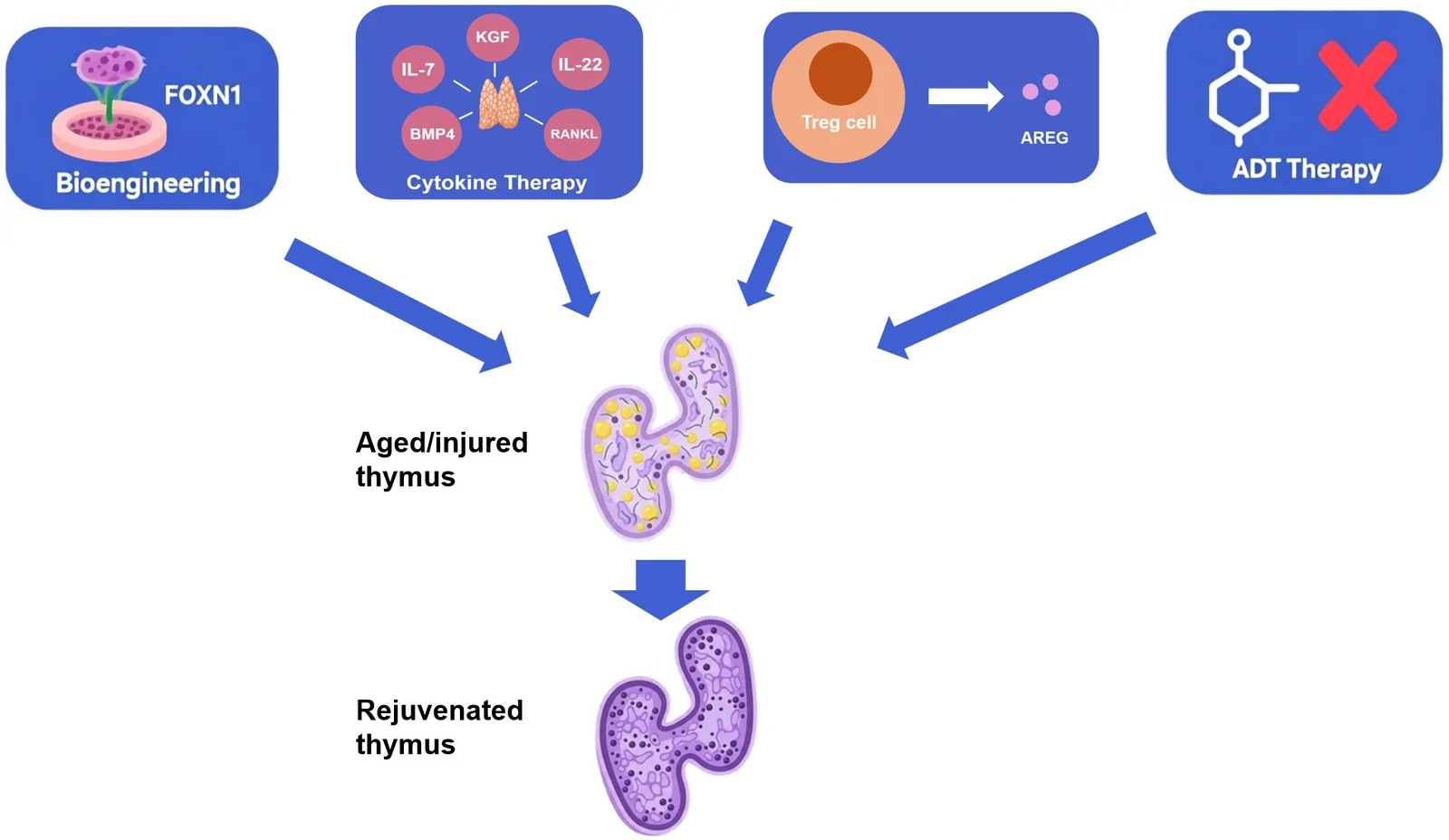
Candidate strategies for thymic regeneration. Bioengineering, cytokine-based approaches, regulatory T Cells and sex-steroid blockade are supported primarily by preclinical or immune-reconstitution studies.

Immunosenescence is closely related to the occurrence and development of various age-related diseases, and thymic involution is a significant cause of immunosenescence. Therefore, restoring thymic function in aged individuals has been a clinical issue of great concern (40, 41). Current strategies to restore patient thymic function are primarily based on studies in mouse experimental models and are founded on: 1) enhancing endogenous thymic regeneration (42); 2) transplanting whole thymic tissue (43); 3) transplanting pluripotent thymic epithelial cells capable of generating a thymic microenvironment *in vivo* and potentially fully restoring thymic function (44). This section summarizes some therapeutic strategies with clinical application prospects, mainly including bioengineering, cytokine, regulatory T Cells and androgen deprivation (**Figure 1**).

### Bioengineering

In recent years, substantial progress has been made in transplantable artificial thymus, which offers an alternative for patients with impaired thymic function and congenital immunodeficiency. Consequently, there is a clinical demand for transplantable thymus tissue, and the bioengineering of a *de novo* thymus *in vitro* represents a promising strategy. Over the past few decades, extensive efforts have been devoted to reconstructing the structure and function of the thymus *in vitro*; however, this field has long been limited by the lack of a sustainable source of epithelial cells.

One research group successfully reprogrammed mouse embryonic fibroblasts into a population of functional thymic epithelial cells (TECs) via forced expression of FOXN1 (45). Following transplantation under the renal capsule of nude mice, these TECs formed an intact and functional thymic organ capable of supporting T cell development. Another team attempted to differentiate human embryonic stem cells into functional TECs to support T cell development (46, 47). In a separate mouse hematopoietic stem cell transplantation model, recombinant FOXN1 protein enhanced thymic T-cell regeneration (48), but this approach has not been validated clinically.

A recent study reported that the implantation of epithelial–mesenchymal hybrid cells and thymic stromal cells into decellularized thymic scaffolds, followed by engraftment into humanized immunodeficient mice, resulted in the formation of a fully structured thymus capable of generating functional T cells (49).

### Cytokine

#### Interleukin-7

Interleukin-7(IL-7) is produced by bone marrow and thymic stromal cells and is critical for the development of T and B cells in mice (50). In the thymus, IL-7 promotes the survival, proliferation, and differentiation of developing T cells. Furthermore, IL-7R mediates cell differentiation via the Jak-Stat signaling pathway and drives cell proliferation and survival through the PI3K/Akt pathway (51). Given the growth-promoting effects of IL-7 on T cells, its role in promoting immune recovery following immune system impairment has been documented. Studies have shown that exogenous IL-7 not only enhances homeostatic proliferation of mature peripheral T cells but also promotes T cell development and the output of recent thymic emigrants (52).

#### Keratinocyte growth factor

Keratinocyte growth factor (KGF) is secreted by mesenchymal cells and primarily promotes epithelial cell proliferation (53). In the thymus, KGF facilitates the proliferation and expansion of medullary thymic epithelial cells (mTECs) mainly by activating the p53 and NF-κB pathways (54), thereby maintaining thymic structural integrity. KGF not only modulates TEC function by downregulating MHC class II molecules involved in positive selection but also regulates cytokines produced by TECs that act directly on thymocytes, including bone morphogenic protein 2 (BMP2), BMP4, Wnt5b, and Wnt10b (55). Studies (56) have demonstrated that exogenous recombinant KGF improves thymic function in young and middle-aged mice and mitigates acute thymic injury induced by therapies such as dexamethasone, cyclophosphamide, and irradiation. These preclinical findings, however, have not translated consistently to humans. In the randomized CAMTHY trial, palifermin administered around alemtuzumab treatment was associated with fewer naive CD4 T cells, recent thymic emigrants (RTEs), and signal joint T-cell receptor excision circles (sjTRECs) than placebo, prompting early termination (57). The investigators hypothesized that KGF might increase CD52 expression on TECs and thereby heighten susceptibility to alemtuzumab-mediated damage, but this mechanism was not directly demonstrated. The result may therefore reflect treatment context, species differences, or a drug interaction and does not support broad use of KGF for thymic regeneration.

#### Bone morphogenetic protein 4

Bone morphogenetic protein 4 (BMP4) acts as a central cytokine governing thymic repair and structural reconstruction following acute injury (42, 58). Predominantly secreted by radioresistant endothelial cells and fibroblasts residing in the thymus, BMP4 serves as an essential signaling molecule that initiates endogenous regenerative programs upon thymic damage. Thymic injury markedly upregulates BMP4 production in endothelial cells. Secreted BMP4 engages BMP receptor type 1A (Bmpr1a) and BMP receptor type 2 (Bmpr2) expressed on thymic epithelial cells (TECs) to trigger downstream signaling cascades (42). Cortical thymic epithelial cells (cTECs) exhibit high levels of functional Bmpr2, rendering them the primary target population of BMP4 (42). Activated BMP4 signaling specifically induces the expression of FOXN1, a master transcription factor governing thymic development, in cTECs and subsequently drives transcriptional activation of its downstream target delta-like ligand 4 (DLL4), thereby establishing the core regenerative BMP4–FOXN1–DLL4 signaling axis (42, 59).

Accumulating evidence has validated that this signaling axis is not only indispensable for the development of thymic epithelial cells and thymocytes (60, 61), but also capable of reversing thymic involution and architectural disorganization elicited by acute injury (42), aging (19) or post-transplant complications (48). It facilitates the proliferation, maturation and functional recovery of thymic epithelial cells to sustain the differentiation, development and reconstitution of thymocytes, augments thymic hematopoietic activity and T cell regenerative capacity, and ultimately restores thymus-dependent immune function *in vivo*. In addition, multiple *in vitro* experiments have verified that BMP4 is tightly involved in the differentiation of multipotent progenitors into thymic epithelial cells, which replenishes the reservoir of functional thymic cells at the stem cell level (46, 47, 62).

Distinct from single regenerative factors, BMP4 simultaneously coordinates thymic structural remodeling and immune functional restoration. It functions as a pivotal regulatory node linking thymic microenvironment repair, TEC turnover and T cell regeneration, representing a promising therapeutic candidate for thymic rejuvenation and the treatment of thymic damage (42).

#### Interleukin-22

As a member of the IL-10 cytokine family, IL-22 typically targets non-hematopoietic cells such as epithelial cells and fibroblasts. Within this regenerative network, acute thymic injury—particularly thymocyte depletion—first triggers dendritic cells to release IL-23, which in turn induces IL-22 production by innate lymphoid cells (ILCs) (63–65). IL-22 then acts on thymic epithelial cells to mediate TEC repair, although the underlying molecular mechanisms remain unclear. Evidence indicates (66) that the IL-22 receptor can induce inactivation of the intracellular Jak1/Tyk2 complex, further promoting STAT3 phosphorylation, which is essential for maintaining thymic epithelial cell homeostasis. A subsequent study linked increased IL-22 expression after thymic injury to elevated Foxn1 expression (67), which may represent an effective pathway for thymic regeneration. Although it is tempting to speculate that IL-22 directly promotes Foxn1 expression, definitive evidence supporting this connection is currently lacking.

#### Regulatory T cells

Regulatory T cells (Tregs) represent a distinct subset of T lymphocytes that exert pivotal functions in sustaining immune homeostasis, preventing autoimmune disorders, and orchestrating the magnitude of immune responses (68). Accumulating prior evidence has documented the involvement of Tregs in wound repair and tissue regeneration across multiple organs, encompassing the heart, liver, kidney, skeletal muscle, lung, bone, and central nervous system (69–79). Recent advances further demonstrate that Treg ablation impairs thymic regeneration by disrupting both thymocyte and stromal compartments, whereas adoptive transfer of Tregs robustly facilitates thymic repair (80).

#### RANKL

Receptor Activator of Nuclear Factor-κB Ligand (RANKL), a member of the tumor necrosis factor (TNF) superfamily, participates in multiple physiological processes in peripheral tissues, with its primary functions centered on pathways governing bone biology (81). RANKL supplied by positively selected CD4 thymocytes contributes to mTEC recovery. In irradiated bone marrow transplantation models, ex vivo RANKL administration expanded TEC subsets, increased thymic homing of lymphoid progenitors, and enhanced *de novo* thymopoiesis (82). This finding provides a mouse proof of concept rather than evidence of an established human therapy.

### Androgen deprivation

Given the impact of sex hormones, especially androgens, on thymic function, surgical or medical castration represents an effective approach to enhancing thymic function (83, 84). Studies have shown that androgen deprivation improves thymic function in both young and aged mice and promotes thymic recovery following immune insults such as chemotherapy or bone marrow transplantation (85, 86). Meanwhile, androgen deprivation therapy (ADT), as a well-established therapeutic modality, is widely used in the clinical management of prostate cancer (87).

In recent years, various chemical agents have been developed to achieve androgen deprivation, acting mainly through three mechanisms: 1)inhibiting androgen synthesis; 2)blocking the binding of androgens to their receptors on target cells; 3)suppressing the hypothalamic–pituitary–gonadal axis to reduce systemic androgen levels. Among these, gonadotropin-releasing hormone agonists (e.g., leuprolide) desensitize gonadotropin-releasing hormone receptors, thereby inhibiting the release of luteinizing hormone and follicle-stimulating hormone, ultimately lowering systemic androgen concentrations. Analyses of prostate cancer patients have demonstrated that leuprolide treatment significantly improves thymic function and increases T-cell output and T-cell receptor excision circle (TREC) levels (83). Another study reported that leuprolide treatment enhances the success rate of hematopoietic stem cell transplantation and promotes T-cell reconstitution in recipients of autologous and allogeneic hematopoietic stem cell transplantation (88).

Although the effects of ADT on thymic regeneration have been investigated for more than a century, the precise mechanisms underlying these regenerative effects remain incompletely understood. Recent studies suggest that androgen deprivation directly upregulates the expression of CCL25 (89) and the Notch ligand DLL4 (90), as well as enhancing the function of hematopoietic stem and progenitor cells (91).

Interestingly, while KGF is not required for ADT-mediated enhancement of thymic function, combination therapy with these two agents shows considerable promise. In a mouse model of bone marrow transplantation, combined administration of KGF and leuprolide attenuated thymic epithelial damage and enhanced T-cell reconstitution (92). These findings support preclinical investigation of sex-steroid blockade for immune reconstitution. Clinical evidence for an antitumor benefit mediated by thymic regeneration, however, remains largely confined to prostate cancer settings in which ADT is already standard therapy. Hematopoietic transplantation studies address T-cell reconstitution rather than tumor control, and the endocrine and metabolic adverse effects of prolonged androgen suppression constrain broad extrapolation to other cancers or patient populations.

## Thymic reconstruction and tumor immunotherapy: evidence and limitations

In recent years, immune checkpoint blockade therapy has played an increasingly important role in the field of cancer treatment. This therapy primarily functions by relieving the inhibitory signals imposed on T cells, activating the immune system, and thereby eliciting effective anti-tumor immune responses. Currently, immune checkpoint inhibitors approved by the FDA mainly target three distinct molecules: CTLA-4, PD-1, and PD-L1, among which PD-1-targeted therapies predominate. Although many patients exhibit significant tumor regression following such treatment, the majority of cancer patients do not respond to these agents (93). Baseline immune competence and intratumoral immune infiltration are among the factors associated with treatment outcome (93). Palmer et al. used epidemiological modeling to propose that age-dependent loss of T-cell production contributes to the rising incidence of several cancers (94). More recently, observational human studies associated better radiographic thymic health with lower all-cause mortality and lung cancer incidence (95), while adult thymectomy was associated with higher mortality and cancer risk (96). Bernatz et al. also reported associations between radiographic thymic health, thymic output, TCR diversity, tumor infiltration, and immunotherapy outcomes across several cancer cohorts (97). Together, these studies identify thymic health as a candidate prognostic biomarker and provide a rationale for testing thymus-directed interventions, but they do not establish that restoring thymic function improves immunotherapy efficacy.

The thymus may influence two related but distinct outcomes: the incidence of new tumors and immune control of established tumors. Reduced production of new T cells can narrow immune surveillance, whereas control of an established tumor also depends on peripheral T-cell function, B cells, NK cells, myeloid cells, and tumor-intrinsic features. Foxn1-deficient mice have severely impaired thymus-dependent T-cell development, whereas Rag2-deficient mice lack mature T and B cells; their tumor phenotypes therefore cannot be treated as an equivalent comparison of thymic function alone. In hyperploid tumor challenge models, different immune-deficient genotypes altered tumor incidence, growth, and immunoselection in distinct ways (98), underscoring the need to analyze these endpoints separately.

The relationship is also bidirectional because cancer itself can alter the thymus. In mouse glioma models, circulating non-steroid soluble mediators drove reversible thymic involution and systemic T-cell loss (99). Cancer cachexia remodeled thymic fibroblasts and impaired negative selection in a hepatocellular carcinoma model (100), while a recent mouse study found that tumor-associated plasmacytoid dendritic-cell subsets can promote thymic deletion of tumor-reactive clones and suppress new T-cell production (101). Direct thymic metastasis from an extrathymic cancer is rare and is supported mainly by case reports (102); its population-level contribution to immune dysfunction is unknown. Cytokine or paraneoplastic effects, cachexia-associated remodeling, direct structural involvement, and treatment-induced injury should therefore be distinguished mechanistically.

**Figure 2:**
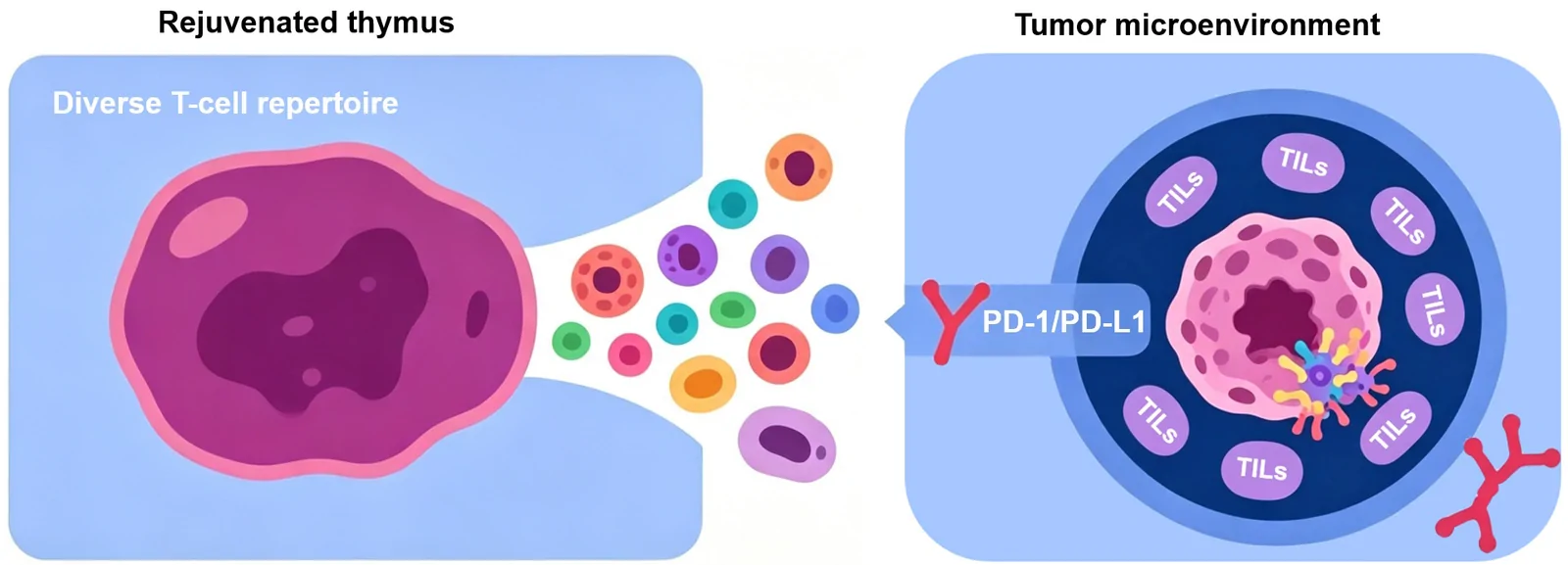
Hypothesized relationship between thymic output, T-cell receptor diversity, and immune checkpoint blockade. Human evidence is observational and does not establish that thymic regeneration improves immunotherapy efficacy.

Recent thymic emigrants are newly generated naive T cells that can replenish peripheral repertoire diversity and potentially introduce new tumor-reactive clonotypes (**Figure 2**). Longitudinal human data demonstrate age-related remodeling and contraction of the peripheral TCR repertoire (103), and recent single-cell profiling has refined phenotypic markers of human RTEs and their age-associated changes (104). Direct antitumor evidence remains preclinical: after androgen blockade in mice, RTEs trafficked to prostate tumors, acquired activation markers, and produced effector cytokines at levels comparable to more mature T cells (105). These data support a testable mechanism but not a proven clinical benefit of increasing RTE output.

Prostate cancer generally has a relatively low tumor mutational burden and limited responsiveness to immune checkpoint blockade (106). Small early-phase studies have evaluated ADT-containing regimens with immunotherapy in this disease. An 11-patient phase I trial of short-term bicalutamide plus tremelimumab focused on safety and observed no favorable early change in PSA doubling time, although delayed prolongation was reported in three patients (107). A randomized phase II study of concurrent versus sequential abiraterone plus prednisone with sipuleucel-T showed preserved product manufacture and immune responses, but it did not test whether ADT improved clinical efficacy relative to immunotherapy alone (108). These studies therefore establish feasibility rather than efficacy enhancement. The clinical antitumor evidence is prostate-cancer-specific, and whether thymic regeneration contributes to treatment outcome remains unproven.

## Conclusion and future perspectives

The thymus is highly susceptible to cytotoxic injury and progressively loses regenerative capacity with age. Restoring thymic output and TCR repertoire diversity therefore provides a biologically plausible strategy for investigation. Human studies now associate radiographic thymic health with health outcomes and immunotherapy response (95, 97). In TRACERx (ClinicalTrials.gov: NCT01888601), higher radiographic thymic health correlated with sjTRECs, peripheral and intratumoral TCR diversity, T-cell infiltration, and adaptive immune signaling (97). Similar associations were observed in several cancer cohorts, but these data are observational; they do not establish causality, a treatment threshold, or benefit from a thymus-directed intervention.

The principal translational limitation is the imbalance between extensive mouse evidence and sparse interventional human evidence. Bioengineered tissues, cytokines, RANKL, Treg-mediated repair, FOXN1 manipulation, and most combinations have not demonstrated clinical antitumor benefit. The discordant KGF trial illustrates how species, dose, timing, disease context, and concomitant therapy can reverse an expected regenerative effect (57). Safety evaluation must also address altered central tolerance and autoimmunity, excessive or off-target growth-factor signaling, expansion of suppressive T-cell populations, and the endocrine and metabolic consequences of sex-steroid blockade. A larger thymus or higher TREC count should not be assumed to indicate a more effective or safer antitumor response.

Future studies should standardize complementary measures of thymic structure and function, including radiographic thymic health, sjTRECs, phenotypic RTEs, naive T-cell output, and TCR diversity. Randomized trials should define target populations, intervention timing, durability, mediation by newly generated T cells, and clinically meaningful endpoints while prospectively monitoring infection, autoimmunity, and cancer outcomes. Until such data are available, established human associations, early translational signals, and preclinical regenerative hypotheses should be reported separately.
